# Are Computed Tomography Scans Necessary for the Diagnosis of Peritonsillar Abscess?

**DOI:** 10.7759/cureus.34820

**Published:** 2023-02-09

**Authors:** Michael J Eliason, Andy S Wang, Jihoon Lim, Richard D Beegle, Michael D Seidman

**Affiliations:** 1 Otolaryngology, Naval Medical Center Portsmouth, Portsmouth, USA; 2 Internal Medicine, Westchester Medical Center, Valhalla, USA; 3 Surgery, University of Central Florida College of Medicine, Orlando, USA; 4 Medical Student, University of Central Florida College of Medicine, Orlando, USA; 5 Radiology, AdventHealth Orlando, Orlando, USA; 6 Otolaryngology, AdventHealth Orlando, Orlando, USA

**Keywords:** diagnostic value of ct scan, peritonsillar cellulitis, peritonsillar phlegmon, peritonsillar abscess, patient safety

## Abstract

Background

Peritonsillar abscess is one of the most common deep-space infections of the head and neck, accounting for significant healthcare costs in the United States. Contributing to this expenditure is the trend of increased usage of computed tomography (CT), particularly in the emergency department. However, CT can be falsely positive for peritonsillar abscess, prompting unnecessary drainage attempts that yield no purulence. The false positive findings question the accuracy of CT in diagnosing peritonsillar abscess.

Objectives

The objective of the study was to compare the accuracy of CT with clinical exam to assess if CT is warranted in peritonsillar abscess diagnosis.

Methods

A retrospective study was performed of patients presenting to eight Orlando emergency departments with throat pain from January 1, 2013, to April 30, 2013. Patients with clinical diagnoses of peritonsillar abscesses were reviewed. A note was made whether CT was performed and if peritonsillar abscess was seen. The reads were compared to the results of procedural intervention for abscess drainage to assess the accuracy of CT in diagnosing peritonsillar abscess.

Results

There were 116 patients diagnosed with peritonsillar abscess, of which 99 underwent CT scans to aid in diagnosis. Among these 99 patients, 23 received procedural intervention, with 16 having a return of purulence (69.6%), and seven remaining without purulence (30.4%).

Conclusion

This study highlights the potential inaccuracies of CT scan in diagnosing peritonsillar abscess, as 30.4% of scans interpreted as abscess lacked purulence on intervention. Given these findings, clinicians could serve as better fiscal stewards by using history and exam to guide management in the majority cases with infectious processes of the oropharynx.

## Introduction

In the second and third centuries B.C., Celcus documented the first known manifestation and treatments of peritonsillar infection. He characterized two varying presentations as swelling without ulceration and edema with obstructed breathing [1]. The infectious process, known today as a peritonsillar abscess (PTA), is now defined as a localized, loculated purulent collection between the capsule of the palatine tonsil and the pharyngeal musculature [2]. PTA is the most common deep-space infection of the head and neck [3,4]. Though PTA can develop in all age groups, it occurs at the highest incidence in adults aged 20-40 years [5]. Culture often demonstrates a polymicrobial infection, but key causative organisms include *Streptococcus pyogenes*, *Fusobacterium necrophorum*, and *Streptococcus milleri* [6,7]. PTA is the most frequent complication of acute tonsillitis resulting in spread to the loose areolar peritonsillar tissues [6,7]. 

Patients with PTA have a fairly classic clinical presentation that otolaryngologists learn early in residency. Clinical symptoms include a severe sore throat, typically unilateral in nature with concomitant dysphagia, odynophagia, radiating otalgia, sialorrhea, systemic fever, and chills. Physical exam findings include marked trismus, inferomedial displacement of the palatine tonsil, contralateral deviation of the uvula, tonsillar erythema and exudate, muffled voice, palatal fullness and fluctuance, and tender cervical lymphadenopathy [3]. It is important to appropriately diagnose and treat PTA to avoid further complications such as spread to adjacent deep neck spaces, the mediastinum, and the skull base [2,6,8-9]. Various treatment algorithms exist, but often drainage of the loculated purulence hastens the patient’s recovery and potentially minimizes further pain, spread of infection, and respiratory obstruction.

In the United States, there are an estimated 30 cases per 100,000 persons per year, accounting for approximately 45,000 cases annually [10,11]. It is estimated that PTA accounts for at least $150 million a year in healthcare expenditures in the United States [10]. Contributing to this significant expenditure is the widespread use of radiographic studies, notably computed tomography (CT) scans, which have seen trends of increased usage particularly in the emergency department (ED) [12]. Despite this increase, studies have estimated that 30% or more of imaging examinations may be unnecessary, costing approximately $30 billion annually in the United States [13]. While many otolaryngologists believe that discerning between a mild form of infection such as cellulitis or phlegmon and an abscess can be readily ascertained based on the aforementioned classic findings, a CT scan is often obtained. Seemingly as part of the triage process by the front-line medical team in the primary, urgent, or emergency care settings, a CT with contrast of the neck is obtained to evaluate for abscess. Anecdotally, this is more common in non-academic settings where they do not have an Otolaryngology- Head and Neck Surgery Residency to readily evaluate patients. Reducing CT use for PTA diagnosis may provide significant savings in healthcare expenditure. While the price of a CT neck with intravenous (IV) contrast can vary significantly per hospital, one study reported an incremental cost-effectiveness ratio of CT neck with IV contrast of $3,306 [14]. Given about 45,000 PTA cases annually and an estimated $3,306 per CT neck with IV contrast, diagnosing PTA clinically without having to order CT scans may save nearly $148 million each year on these studies (45,000 PTA cases x $3,306 for CT neck with IV contrast = $148,770,000) [10,11,14].

The addition of the CT not only adds significant economic cost, but also contributes to additional potential harm of radiation exposure and IV contrast for the patient. A routine CT imaging (120kVP) of the head and neck results in a radiation dose of 2.1-3.9 mSv [15]. While low-dose CT (80kVP) have been used to enhance PTA diagnosis and address these concerns, ionizing radiation from either doses carry risks of malignancies and other adverse effects [16-17]. Furthermore, it remains unclear whether CT accurately differentiates phlegmon from abscess [18]. Therefore, the objective of this study was to compare the accuracy of CT to clinical exam to determine if clinical exam alone is sufficient, thereby reducing the need for CT and lowering radiation exposure and significant healthcare expenses.

## Materials and methods

After obtaining approval from the Institutional Review Board of Central Florida, a retrospective chart review was performed of all patients who presented with throat pain (International Classification of Diseases, Ninth Revision (ICD-9): 462, 463, 475, and 784.1) to one of eight Orlando area EDs between January 1, 2013, and April 30, 2013.

Electronic medical records from over 6,000 patients met inclusion for review. Each chart was reviewed by clinical staff to determine if the patient met the criteria by the clinical staff for the ultimate clinical diagnosis of PTA. Symptoms and exam findings for clinical diagnosis included unilateral sore throat, dysphagia, odynophagia, otalgia, fever, trismus, muffled voice, tender cervical lymphadenopathy, tonsillar erythema and exudate, uvula deviation, tonsillar displacement, peritonsillar bulging, and palatal fullness and fluctuance [3,19]. The mean age of the patients reviewed was 28.6 years old. Pediatric patients under 18 years old were excluded. A note was made whether a CT neck with IV contrast was performed to aid in diagnosis. Findings suggestive of PTA on CT included a rim-enhancing collection typically superolateral to an enlarged palatine tonsil [19,20]. Patients were excluded if they received a non-contrast CT scan of the neck. The reads of these CTs were reviewed to determine if a PTA diagnosis was made by the radiologist.

The radiology reads were compared to the results of a procedural intervention for abscess drainage in the applicable patients, which included needle aspiration, incision and drainage (I&D), and surgical tonsillectomy. Specifically, documented return of purulence by the surgeon was considered necessary to confirm the diagnosis of PTA. On the contrary, aspiration or drainage attempts without purulent return were classified as phlegmonous or cellulitis infections. Patients who did not undergo procedural intervention received medical management only, which included antibiotic treatment with or without systemic corticosteroid therapy.

## Results

**Figure 1:**
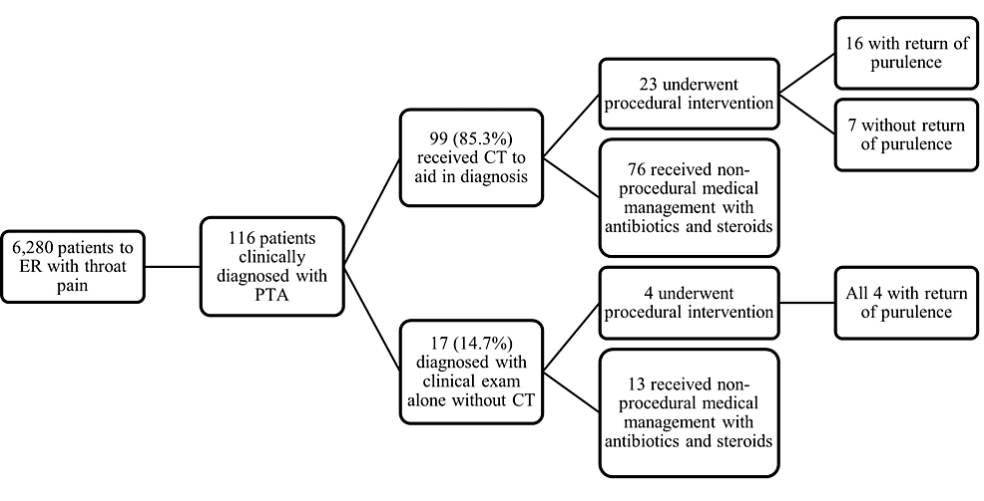
Flow Chart of the Distribution of Patients. Breakdown of patients who were clinically diagnosed with PTA, received CT to aid in diagnosis, and underwent procedural intervention or non-procedural medical management

**Figure 2:**
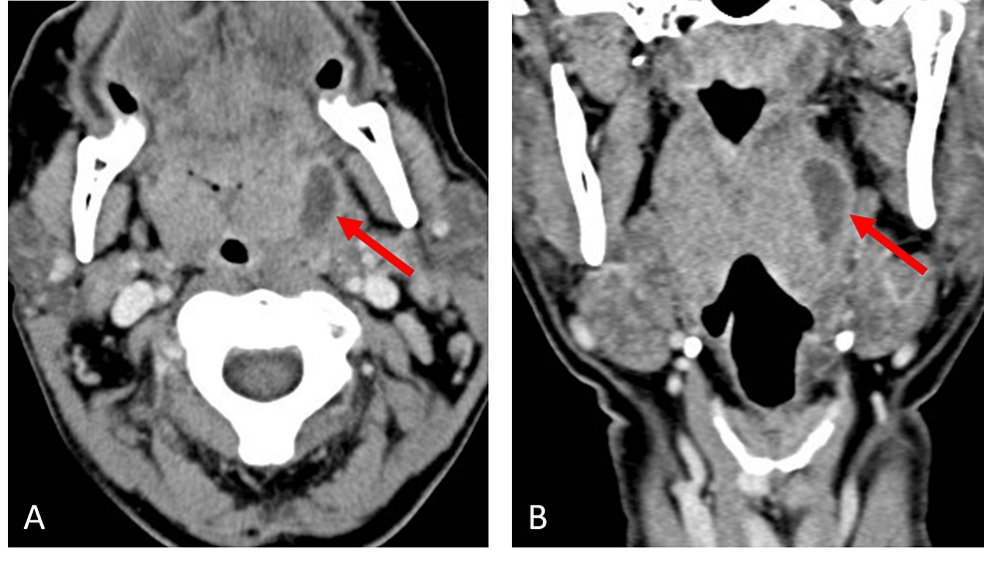
Peritonsillar Abscess Axial (A) and Coronal (B) View CT with contrast images demonstrating enlarged bilateral palatine tonsils and peripherally enhancing fluid collection in the left parapharyngeal space consistent with peritonsillar abscess (arrow).

**Figure 3:**
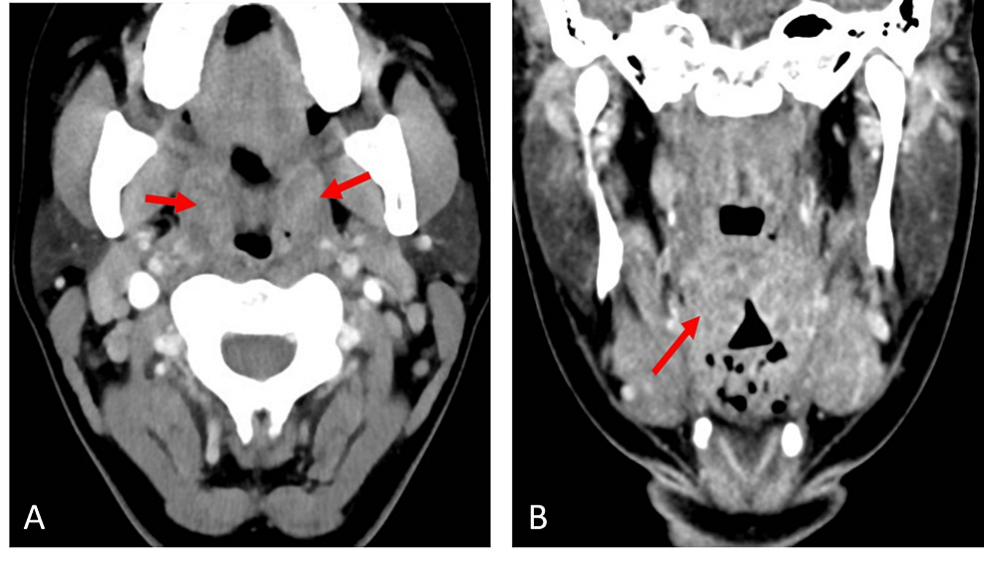
Tonsillitis Axial (A) and Coronal (B) View CT with contrast images demonstrate typical findings of tonsillitis to include bilateral tonsillar enlargement with striated internal enhancement (arrows) consistent with early intratonsillar phlegmonous changes. This scan is negative for a well-defined peripherally enhancing abscess.

A total of 6,280 patients met inclusion for review during the time frame examined and 116 of these were clinically diagnosed with PTA; though of note, there is only an annual incidence of 30 cases of PTA per 100,000 persons in the United States [5,10,11]. The mean age of the patients reviewed was 28.6 years old; of note, Galioto et al. report that while PTA can develop at all ages, the highest incidence is in adults aged 20 to 40 years [5]. As demonstrated in Figure 1, among the 116 who were clinically diagnosed with PTA, 85.3% (n=99) underwent radiographic assessment via CT. Findings on CT suggestive of PTA included enlarged palatine tonsils with adjacent peripherally enhancing fluid collection, as seen on the axial and coronal CT in Figure 2. In comparison, prior to the development of PTA, patients may initially present with signs of tonsillitis, cellulitis, and phlegmon, as seen on the axial and coronal CT of Figure 3, which shows findings of tonsillitis with enlarged palatine tonsils, striated internal enhancement pattern, and phlegmonous changes, but no peripherally enhancing collection as seen in PTA. Only 14.7% (n=17) were diagnosed based on clinical exam alone. Of those diagnosed with PTA, 23.3% (n=27) underwent procedural intervention by the treatment team. The remaining 76.7% (n=89) patients were treated with medical management and did not have procedural intervention. Figure 1 delineates this clinical decision-making by the front-line triage team in the diagnosis and management of PTA.

**Figure 4:**
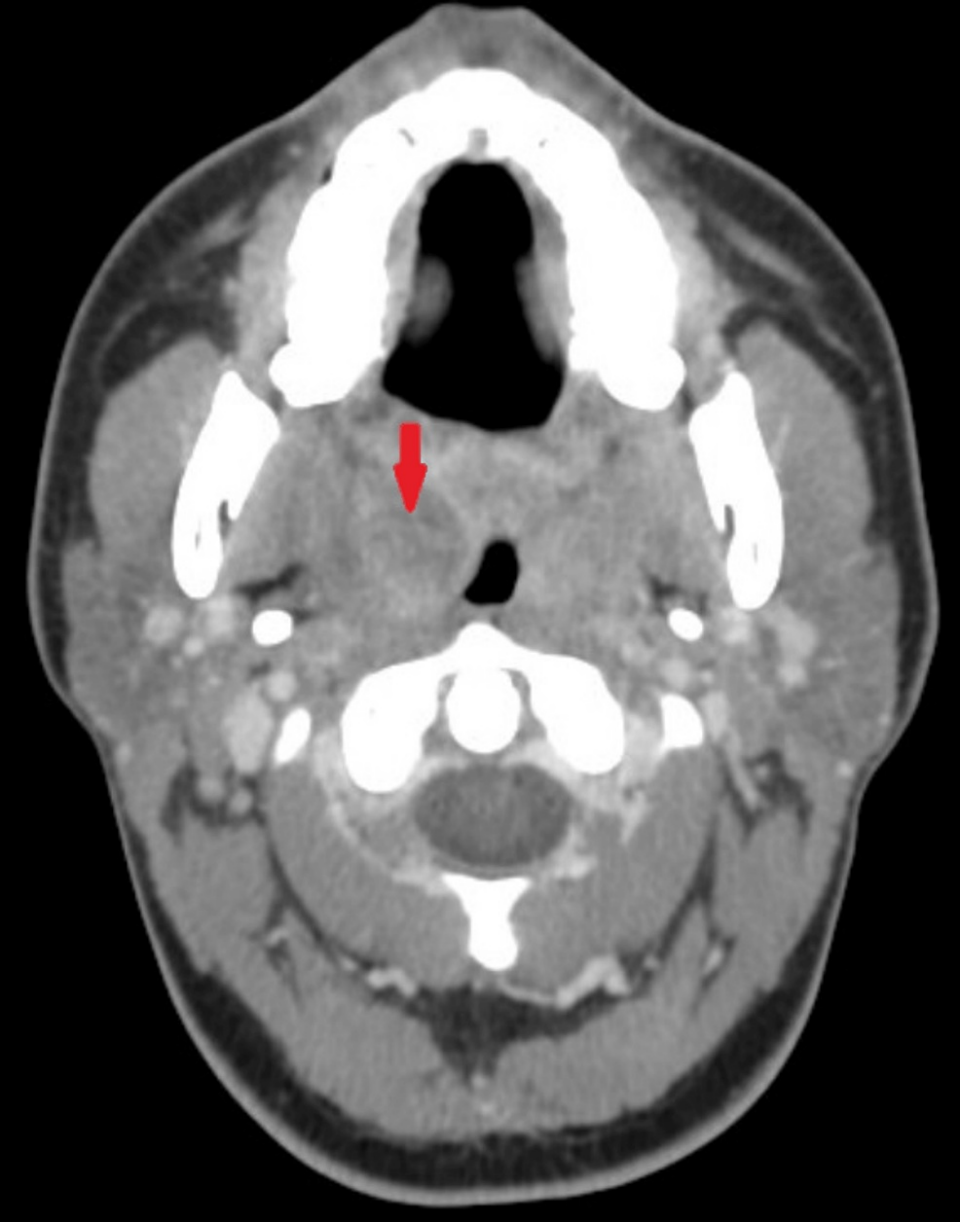
Peritonsillar Phlegmon Axial CT with contrast demonstrating findings in the right peritonsillar region (arrow) that was interpreted as a likely abscess prompting otolaryngology procedural intervention, though multiple aspiration attempts did not return purulence. Findings were most consistent with phlegmonous changes, highlighting the potential inaccuracy of CT in discerning abscesses from phlegmon.

As seen in Figure 1, among the 99 patients who received radiographic assessment with CT for PTA, 23 underwent procedural intervention, with purulence confirmed in 69.6% of patients (n=16). The other 30.4% (n=7) who underwent procedural intervention without return of purulence either fell into the category of peritonsillar phlegmon and cellulitis (i.e., misdiagnosed by CT as an abscess), or likely had an abscess that was too small or loculated for return of purulence during intervention. For instance, Figure 4 shows an axial CT that was interpreted as a possible abscess, leading to otolaryngology intervention, though drainage attempts did not yield pus, with findings instead suggestive of phlegmonous changes, demonstrating possible CT inaccuracy in differentiating abscess from phlegmon. The remaining 76 out of 99 patients who underwent CT received non-procedural medical management with antibiotics and steroids.

Clinical diagnosis of PTA without CT imaging was made in 14.7% of the 116 PTA patients (n=17). Among these 17 patients, four (23.5%) underwent procedural intervention with I&D with return of purulence and confirmed PTA in all patients. The remaining 13 patients (76.5%) of those diagnosed clinically underwent medical management with antibiotics with or without corticosteroids.

A secondary outcome assessed the relative percentage of each type of procedural intervention for the 27 patients that underwent procedures. Bedside procedures using a local anesthetic (needle aspiration or I&D) represented 85.2% (n=23) of the total procedures. Surgical tonsillectomy accounted for 14.8% (n=4).

## Discussion

Early diagnosis and treatment of PTA are paramount to hasten patient recovery and minimize the potential for morbid complications. However, the trend toward the utilization of CT scans to diagnose what many otolaryngologists deem a clinical diagnosis likely results in wasted resources and the potential for long-term patient harm from unnecessary radiation exposure. Perhaps adding to the pressure of utilizing more CT scans is the desire of consulting physicians in the ED or other front lines of healthcare to have an objective test beyond their clinical exam before calling for a surgical consultation. Especially in geographic locations without residency training programs, it is often easier to call in a consult when you have a test that confirms it is necessary.

These data comparing diagnosis with CT to a procedural intervention demonstrate that 30.4% of patients had a scan that stated there was purulence, but none was found. While a procedure negative for purulence does not absolutely rule out a smaller abscess or poorly performed procedure, it does question the accuracy of diagnosing a PTA that requires intervention. An example is the scan depicted in Figure 4 of one of the subjects from this study. This axial CT was interpreted as a likely abscess in the right peritonsillar region prompting referral for procedural intervention. However, this patient’s clinical exam and multiple aspiration attempts did not return purulence and were most consistent with phlegmonous changes. These cases represent the classic conundrum experienced by an otolaryngologist called to the ED; the CT ordered by the emergency physician shows a PTA, thereby motivating a perceived necessary intervention only to result in an unsuccessful drainage attempt and treatment ultimately with medical management. This case highlights the potential inaccuracy of CT in discerning abscesses from phlegmons, leading to false positive detections of PTA. These false positive findings may be explained by a study from Capps et al., which noted that CT is early 100% sensitive and approximately 75% specific for PTA detection, suggesting that while a negative CT is helpful for ruling out PTA given its high sensitivity, a positive CT may not be helpful for detecting PTA as it is likelier to yield a false positive given its lower specificity [19-21]. A critical point is that diagnosis can often be made by examination of the oropharynx, obviating the need for radiographic imaging.

Distinguishing between an abscess and phlegmonous changes can prove difficult in the oropharynx on CT. An abscess is identified radiographically as a well-defined enhancing capsule with internal hypodensity approaching fluid density (<20 Hounsfield units). Phlegmon represents inflammation that appears typically as an area of relative hypodensity compared to the surrounding enhancing inflammation and tonsil, but will not have a well-defined capsule. There is certainly a spectrum where the phlegmon may transition to an abscess, and depending on the timing of the radiograph, it may be difficult to discern.

This correlates clinically to patients who may present initially with tonsillitis, which can progress to cellulitis, phlegmonous changes, and eventually into PTA or tonsillar abscess. Figure 3 demonstrates classic radiographic findings of tonsillitis on axial and coronal CT including bilaterally enlarged palatine tonsils with a heterogeneous striated internal enhancement pattern, intratonsillar phlegmonous changes, and reactive lymphadenopathy, but no well-defined peripherally enhancing abscess.

This is contrasted to the PTA seen in the axial and coronal CT in Figure 2, which demonstrates similarly enlarged bilateral palatine tonsils with increased enhancement, as well as well-defined peripherally enhancing fluid collection in the left parapharyngeal space, potentially displacing the tonsil medial and contributing to aerodigestive tract symptoms. These figures demonstrate classic radiographs as the infection progresses from tonsillitis to abscess but, as the results from this study demonstrate, the findings are often somewhere in between and can misguide further intervention.

Recent literature has supported the use of clinical exam alone to properly diagnose PTA. In fact, recent studies on symptom data-driven algorithms and even telehealth consultation have found them to be reliable means to properly diagnose PTA and assess the need for procedural intervention [22,23]. These studies noted symptoms like otalgia, trismus, duration of symptoms, neck pain, worsening of symptoms, and no previous treatment to be adequate directors of procedural intervention. A study by Mallen et al. showed reasonable reliability in clinical history and a smartphone video of the oropharyngeal exam alone to direct otolaryngologic intervention [22]. Furthermore, multiple pediatric studies have demonstrated that an otolaryngology consult for clinical exam prior to imaging significantly reduces the frequency of CT scans ordered and a higher yield for intervention in those obtained [24,25]. The utilization of smartphones provides other avenues of PTA diagnosis. Ban et al. reported an effective diagnosis of PTA using smartphone-based thermography as a noncontact and noninvasive method to detect significant differences in abscess temperature [26].

Ultrasonography (US) can be considered a separate imaging modality as an alternative to CT [27]. Studies have demonstrated that intraoral US can accurately diagnose PTA and assist in the safe drainage of abscesses, which can help eliminate the risk of blind needle aspirations [27]. Intraoral US has been reported to have a sensitivity of 89-95% and a specificity of 78-100% [28]. In addition, Fordham et al. reported the sensitivity and specificity of transcervical US in 43 pediatric patients as 100% and 76.5%, respectively [28]. US can elucidate the heterogeneous and cystic structures of PTA and has the potential to reliably differentiate PTA from peritonsillar cellulitis in clinically equivocal presentations, thereby resulting in less unnecessary needle aspiration, lower CT usage, and fewer otolaryngology consults [12,26,27].

Despite the potential benefit of US or CT, it may be more reasonable and cost-effective to rely on clinical exam alone. There may be a limited need for additional imaging in the evaluation of patients with pharyngitis, tonsillitis, phlegmon, or abscess. Clinical acumen should generally be the primary means of diagnosis.

Efficiency and respect for timeliness have become paramount across the healthcare system, as late discharges are thought to be associated with overcrowding and increased length of stay [29]. Multiple reward mechanisms are in place to encourage the timely treatment and discharge of patients from urgent and emergent care facilities [29]. Wertheimer et al. reported earlier discharges and reduced readmission rates at two acute units in an academic medical center after implementing an intervention that included education and discussion on the importance of safe early discharges, discharge responsibility checklists, daily feedback on discharge times and rates, and a reward system if discharge goals were met [29]. This has therefore resulted in diagnostic and therapeutic options that are quicker, but likely also to incur risk. One such example is the use of CT or US imaging for the diagnosis of PTA. A period of observation to ensure improvement of symptoms after the initial dosing of antibiotics and steroids takes time and valuable patient bed and space where other patients may be seen and treated. Thus, healthcare systems have grown to favor a quick imaging study that can more efficiently dictate whether a surgeon’s consultation and procedural intervention may be required.

Some posit that imaging to catch the non-obvious clinical PTA is unnecessary. Specifically, while still a controversial topic amongst otolaryngologists, some recent reviews have indicated good prognoses with medical management alone, or at least initially, even for PTA with likely purulence [2,30]. Battaglia et al. demonstrated that treatment with an algorithm of one-time IV antibiotics and steroids while in the ED followed by an outpatient course of clindamycin reduces the possibility of procedural complications, cost, and inconvenience [30]. The concept of medical management alone as a treatment for a potentially small loculated peritonsillar infection may further support a “no-scan” approach or detract from the need for imaging in the diagnosis. These studies suggest that a trial of antibiotics with or without corticosteroids may be sufficient for the treatment of smaller PTA, and imaging in the acute setting is therefore unlikely to change management. In these patients who are clinically borderline, meaning they may have a small abscess vs phlegmon, a trial of medical management may be appropriate. In patients who are beyond "borderline" and have obvious purulent collections for which acute procedural intervention is necessary based on physical exam, a CT is unlikely to aid in the clinical decision-making.

Generally, the main procedure interventions used to treat PTA include needle aspiration, I&D, and tonsillectomy [3]. Multiple studies have demonstrated that all three procedures are equally effective in treating PTA with no statistically significant differences in overall outcomes [5]. I&D and needle aspiration are typically favored over immediate tonsillectomy in the acute setting [3,5]. The timing of tonsillectomy remains controversial. One study noted that immediate tonsillectomy can be considered in children and patients with recurrent episodes of tonsillitis and previous PTAs since they may be at a higher risk of recurrence [5]. However, other studies report that immediate tonsillectomy carries an elevated risk of intraoperative bleeding and sepsis from operating during an ongoing infection, and that tonsillectomy should instead be deferred until three to six months after the abscess in patients with a history of recurrent PTA or tonsillitis [3].

While CT is not generally obtained when PTA is clinically apparent, there are some circumstances where CT neck with IV contrast may be helpful. CT may be considered if a thorough clinical exam cannot be performed (i.e., unable to fully assess the oropharynx if a patient has severe trismus), if the diagnosis is ambiguous and alternative diagnoses are suspected, if the patient is unresponsive to treatment, or if there is suspicion for complications (i.e., spread beyond peritonsillar space into surrounding spaces, including masticator space) or other deep neck space infections (i.e., retropharyngeal abscess) [19]. Additionally, in the emergency setting, a CT neck can be obtained to evaluate symptoms of aerodigestive or vascular compromise to assess for patency, particularly when symptoms are suspected to be related to the neck, though airway management should be prioritized in the setting of a tenuous airway and should not be delayed for a CT [20]. If possible, PTA should be distinguished from intratonsillar abscess, as the latter may not require procedural intervention to drain the abscess [20]. Nevertheless, it is crucial to emphasize that given CT neck with IV contrast is nearly 100% sensitive and 75% specific for PTA, there may be false positive findings given the challenge of discerning PTA from phlegmon, so caution is needed when using CT to aid in diagnosis [19,20].

Limitations of this study are the result of its retrospective nature that precludes standardization of diagnosis or intervention. This retrospective study lacks the ability to follow these patients longitudinally to assess for outcomes. A prospective study could randomize patients to diagnostic and treatment algorithms to elucidate what is ultimately best for patient outcomes. Additionally, while it is customary for a surgeon to make multiple passes in the peritonsillar region, the authors recognize it is entirely possible that a small loculated region of purulence could be missed. Further, given the annual incidence of PTA is 30 cases per 100,000 persons in the United States, one limitation is the small sample size, especially since only 27 patients underwent intervention, so disclosing the true accuracy of CT may be limited [5,10,11]. Future studies will involve recruiting a larger sample size of patients with PTA who undergo procedural intervention to better compare the accuracy of CT to clinical exam alone.

## Conclusions

Given 99 out of 116 patients clinically diagnosed with PTA in our study also received CT to aid in diagnosis, this could suggest high frequency usage of CT scan for the diagnosis of PTA by front-line provider teams in the ED and primary care setting. This study highlights the difficulty in discerning oropharyngeal phlegmon from abscess on a CT, as the data demonstrated approximately 30% of cases where a CT was read as positive for abscess but a procedural attempt at drainage was unsuccessful at finding purulence. The authors propose that given the study’s findings, clinicians could serve as better fiscal stewards while also avoiding potential negative effects of imaging by using the clinical history and physical exam to guide management in the majority of those presenting with an infectious process of the oropharynx. However, the authors also acknowledge that CT may be helpful is certain instances and can be considered when a full clinical exam cannot be performed, if alternative diagnoses are suspected, if there is poor response to treatment, when there is suspicion for complications or other deep neck space infections, or if aerodigestive and vascular compromise are present, though airway management should not be delayed to perform a CT. For future research, a prospective study is planned, which will involve preparing a clinical training opportunity in the ED that highlights the differences between pharyngitis, peritonsillar phlegmon, and PTA, and assessing if the clinical training can help to reduce the ordering of CT scans. 
